# Supplementary material for: Variability of enteric pathogen infections by season and meteorological conditions in a low-income, urban setting in Mozambique
Source: PLOS Glob Public Health. 2026 Apr 28;6(4):e0005330. doi: 10.1371/journal.pgph.0005330 (PMC13123936; doi:10.1371/journal.pgph.0005330)
Supplement: S1 Table — (PDF) [file pgph.0005330.s001.pdf]

S1 Table. Study population characteristics (N=630)

	**n (%) or Mean (SD)**
Female index child	302 (47.9%)
High poverty based on socio-economic status*	383 (60.8%)
Caregiver completed at least secondary education*	151 (24.0%)
Primary caregiver has fixed employment*	229 (36.3%)
Number of children under 5 living in household*	1.4 (0.6)
Number of people in the household*	5.6 (2.5)
Months living in the household*	69.7 (80.8)
Human feces observed in or near the household*	6 (1.0%)
Animal feces observed in or near the household*	84 (13.3%)
Severely food insecure*	297 (47.1%)
Handwashing station in household or yard at baseline	129 (20.5%)
Basic household sanitation access at baseline	226 (35.9%)
Improved water	630 (100.0%)
Drinking water source on premises	305 (48.4%)
Water insecure (HWISE) at baseline	86 (13.8%)

* Data are reported for 12-month visit whereas they are reported at baseline for the main PAASIM analysis
