# Supplementary material for: Variability of enteric pathogen infections by season and meteorological conditions in a low-income, urban setting in Mozambique
Source: PLOS Glob Public Health. 2026 Apr 28;6(4):e0005330. doi: 10.1371/journal.pgph.0005330 (PMC13123936; doi:10.1371/journal.pgph.0005330)
Supplement: S2 Table — (PDF) [file pgph.0005330.s003.pdf]

**S2 Table.** Adjusted associations of Heavy Rainfall Events (HREs) on enteric pathogen infections.

| | **0-1 week before sample** | | **1-2 weeks before sample** | | **2-3 weeks before sample** | |
|---|---|---|---|---|---|---|
| | **aPR or aβ (95% CI)** | ***p-value*** | **aPR or aβ (95% CI)** | ***p-value*** | **aPR or aβ (95% CI)** | ***p-value*** |
| **Combined outcomes** | | | | | | |
| **Any bacteria** | 0.98 (0.88, 1.08) | 0.64 | 1.01 (0.92, 1.10) | 0.85 | 0.99 (0.88, 1.11) | 0.82 |
| **Any protozoa** | 0.93 (0.71, 1.22) | 0.61 | 1.30 (1.06, 1.59) | 0.01 | 0.99 (0.79, 1.24) | 0.93 |
| **Any virus** | 1.24 (0.97, 1.60) | 0.09 | 1.22 (0.95, 1.57) | 0.12 | 0.96 (0.75, 1.22) | 0.73 |
| **Co-infection** | 1.05 (0.92, 1.20) | 0.44 | 1.09 (0.99, 1.21) | 0.09 | 0.99 (0.84, 1.17) | 0.91 |
| **Number of infections** | -0.12 (-0.32, 0.08) | 0.25 | -0.04 (-0.22, 0.14) | 0.63 | -0.01 (-0.24, 0.21) | 0.91 |
| **Bacterial outcomes** | | | | | | |
| **EAEC** | 1.03 (0.85, 1.24) | 0.78 | 0.88 (0.72, 1.08) | 0.23 | 1.09 (0.89, 1.33) | 0.40 |
| **DAEC** | 0.92 (0.82, 1.03) | 0.13 | 1.03 (0.96, 1.12) | 0.42 | 0.94 (0.85, 1.04) | 0.24 |
| **tEPEC** | 0.85 (0.63, 1.14) | 0.27 | 0.94 (0.66, 1.32) | 0.71 | 1.01 (0.63, 1.60) | 0.98 |
| **aEPEC** | 1.06 (0.87, 1.29) | 0.56 | 1.01 (0.79, 1.30) | 0.93 | 0.97 (0.72, 1.32) | 0.86 |
| **ETEC** | 0.83 (0.51, 1.34) | 0.44 | 0.41 (0.23, 0.73) | 0.00 | 0.99 (0.58, 1.68) | 0.96 |
| **Shigella** | 0.90 (0.61, 1.33) | 0.60 | 0.80 (0.56, 1.16) | 0.24 | 1.01 (0.68, 1.50) | 0.95 |
| **Campylobacter** | 0.86 (0.63, 1.18) | 0.35 | 1.23 (0.92, 1.66) | 0.17 | 1.04 (0.73, 1.47) | 0.85 |
| **Viral outcomes** | | | | | | |
| **Norovirus** | 1.62 (1.02, 2.58) | 0.04 | 1.12 (0.68, 1.86) | 0.65 | 0.73 (0.4, 1.33) | 0.30 |
| **Protozoan outcomes** | | | | | | |
| **Cryptosporidium** | 0.70 (0.43, 1.14) | 0.15 | 1.41 (0.98, 2.02) | 0.06 | 1.15 (0.80, 1.65) | 0.46 |
| **Giardia** | 1.12 (0.75, 1.68) | 0.57 | 1.12 (0.84, 1.50) | 0.45 | 0.93 (0.66, 1.31) | 0.67 |

HREs were defined as a day where the total rainfall was above the 95$^{th}$ percentile (8.81mm) for the overall study period. All models adjusted for rolling mean temperature during the same period, intervention status, access to a direct household connection to a piped water source, poverty, caregiver education level, caregiver employment status, and basic sanitation access. Models for specific infection only run for enteric infections with prevalence over 10%.
