# Supplementary material for: Variability of enteric pathogen infections by season and meteorological conditions in a low-income, urban setting in Mozambique
Source: PLOS Glob Public Health. 2026 Apr 28;6(4):e0005330. doi: 10.1371/journal.pgph.0005330 (PMC13123936; doi:10.1371/journal.pgph.0005330)
Supplement: S3 Table — (PDF) [file pgph.0005330.s004.pdf]

S3 Table. Sensitivity analysis of associations between Heavy Rainfall Events (HREs) and enteric infections using 80th and 90th percentile HRE cutoffs

	0-1 week before sample		1-2 weeks before sample		2-3 weeks before sample	
	**aPR or aβ (95% CI)**	***p-value***	**aPR or aβ (95% CI)**	***p-value***	**aPR or aβ (95% CI)**	***p-value***
**90th Percentile**						
**Combined outcomes**						
**Any bacteria**	0.93 (0.85, 1.03)	0.15	0.93 (0.86, 1.01)	0.10	1.01 (0.90, 1.14)	0.81
**Any protozoa**	1.00 (0.79, 1.26)	0.98	1.17 (0.96, 1.43)	0.11	0.99 (0.83, 1.17)	0.89
**Any virus**	1.13 (0.91, 1.41)	0.26	1.15 (0.93, 1.41)	0.19	0.94 (0.76, 1.16)	0.54
**Co-infection**	0.99 (0.88, 1.11)	0.83	1.01 (0.90, 1.14)	0.85	1.05 (0.89, 1.24)	0.58
**Number of pathogens**	-0.13 (-0.34, 0.08)	0.22	-0.17 (-0.34, 0.00)	0.08	0.02 (-0.20, 0.24)	0.85
**Bacterial outcomes**						
**EAEC**	0.96 (0.82, 1.14)	0.65	0.99 (0.83, 1.17)	0.89	0.97 (0.80, 1.18)	0.74
**DAEC**	0.88 (0.79, 0.97)	0.01	0.94 (0.86, 1.02)	0.15	0.94 (0.87, 1.03)	0.18
**tEPEC**	0.77 (0.59, 0.99)	0.04	0.89 (0.70, 1.14)	0.35	0.87 (0.60, 1.28)	0.49
**aEPEC**	0.97 (0.80, 1.19)	0.79	0.98 (0.79, 1.22)	0.88	1.19 (0.94, 1.51)	0.14
**ETEC**	1.08 (0.71, 1.63)	0.74	0.41 (0.26, 0.66)	0.00	0.72 (0.47, 1.10)	0.12
**Shigella**	0.95 (0.70, 1.30)	0.76	0.69 (0.49, 0.99)	0.05	1.06 (0.70, 1.61)	0.77
**Campylobacter**	0.91 (0.70, 1.18)	0.46	1.18 (0.91, 1.53)	0.22	1.02 (0.76, 1.36)	0.90
**Viral outcomes**						
**Norovirus**	1.44 (0.93, 2.21)	0.10	1.17 (0.74, 1.84)	0.51	0.97 (0.64, 1.46)	0.87
**Protozoan outcomes**						
**Cryptosporidium**	0.79 (0.52, 1.20)	0.26	1.17 (0.80, 1.70)	0.41	1.03 (0.79, 1.34)	0.84
**Giardia**	1.10 (0.75, 1.63)	0.63	1.00 (0.77, 1.30)	0.99	1.01 (0.79, 1.29)	0.95
**80th Percentile**						
**Combined outcomes**						
**Any bacteria**	0.96 (0.85, 1.08)	0.47	0.92 (0.83, 1.03)	0.15	0.96 (0.87, 1.06)	0.42
**Any protozoa**	0.77 (0.61, 0.96)	0.02	0.91 (0.72, 1.16)	0.45	0.99 (0.82, 1.20)	0.93
**Any virus**	0.97 (0.79, 1.20)	0.80	1.14 (0.90, 1.45)	0.28	0.91 (0.72, 1.14)	0.39
**Co-infection**	0.88 (0.75, 1.04)	0.13	0.94 (0.82, 1.08)	0.38	0.96 (0.83, 1.10)	0.53
**Number of pathogens**	-0.21 (-0.45, 0.04)	0.10	-0.15 (-0.36, 0.05)	0.14	-0.04 (-0.26, 0.17)	0.67
**Bacterial outcomes**						
**EAEC**	0.95 (0.83, 1.10)	0.52	0.87 (0.75, 1.02)	0.08	0.84 (0.67, 1.05)	0.12
**DAEC**	0.93 (0.85, 1.02)	0.14	0.93 (0.84, 1.03)	0.16	0.95 (0.86, 1.05)	0.31

**tEPEC**	0.89 (0.64, 1.22)	0.46	0.85 (0.61, 1.20)	0.36	0.72 (0.49, 1.05)	0.09
**aEPEC**	0.84 (0.67, 1.06)	0.14	1.00 (0.79, 1.27)	1.00	1.14 (0.87, 1.48)	0.35
**ETEC**	0.92 (0.58, 1.44)	0.71	0.60 (0.34, 1.07)	0.08	0.76 (0.45, 1.26)	0.28
**Shigella**	1.05 (0.68, 1.62)	0.82	0.90 (0.62, 1.33)	0.60	0.95 (0.65, 1.37)	0.77
**Campylobacter**	1.02 (0.75, 1.38)	0.93	1.08 (0.79, 1.47)	0.63	0.92 (0.68, 1.24)	0.58
**Viral outcomes**						
**Norovirus**	1.20 (0.78, 1.86)	0.41	1.23 (0.76, 2.01)	0.40	1.12 (0.72, 1.73)	0.62
**Protozoan outcomes**						
**Cryptosporidium**	0.65 (0.46, 0.92)	0.02	0.91 (0.62, 1.33)	0.63	1.11 (0.80, 1.56)	0.54
**Giardia**	0.83 (0.59, 1.18)	0.30	0.88 (0.65, 1.20)	0.42	1.05 (0.77, 1.44)	0.74
