# Supplementary material for: Variability of enteric pathogen infections by season and meteorological conditions in a low-income, urban setting in Mozambique
Source: PLOS Glob Public Health. 2026 Apr 28;6(4):e0005330. doi: 10.1371/journal.pgph.0005330 (PMC13123936; doi:10.1371/journal.pgph.0005330)
Supplement: S4 Table — (PDF) [file pgph.0005330.s005.pdf]

S4 Table. Adjusted associations of Heavy Rainfall Events (HREs) on enteric pathogen infections by enteric pathogen infections including interaction by antecedent conditions.

	**0-1 week before sample**		**1-2 weeks before sample**		**2-3 weeks before sample**	
	**aPR or aβ (95% CI)**	***p-value***	**aPR or aβ (95% CI)**	***p-value***	**aPR or aβ (95% CI)**	***p-value***
**Any Bacteria**						
**Wet**	0.96 (0.82, 1.11)	0.57	1.06 (0.92, 1.23)	0.40	1.01 (0.87, 1.18)	0.89
**Medium/Dry**	1.01 (0.77, 1.34)	0.92	0.98 (0.76, 1.25)	0.86	0.98 (0.75, 1.28)	0.88
**Interaction**	1.06 (0.84, 1.34)	0.63	0.92 (0.75, 1.12)	0.40	0.97 (0.78, 1.20)	0.78
**Any Protozoa**						
**Wet**	0.89 (0.59, 1.34)	0.57	1.34 (0.94, 1.93)	0.11	1.09 (0.74, 1.60)	0.67
**Medium/Dry**	0.94 (0.49, 1.84)	0.87	1.32 (0.72, 2.41)	0.37	0.85 (0.41, 1.76)	0.66
**Interaction**	1.06 (0.63, 1.79)	0.82	0.98 (0.60, 1.59)	0.94	0.78 (0.42, 1.45)	0.44
**Any Virus**						
**Wet**	1.48 (1.12, 1.94)	0.01	1.24 (0.95, 1.63)	0.12	1.30 (0.97, 1.74)	0.08
**Medium/Dry**	1.03 (0.59, 1.80)	0.92	1.25 (0.79, 1.98)	0.34	0.64 (0.35, 1.16)	0.14
**Interaction**	0.70 (0.43, 1.13)	0.15	1.01 (0.69, 1.46)	0.97	0.49 (0.29, 0.83)	0.01
**Co-infection**						
**Wet**	1.02 (0.85, 1.23)	0.82	1.14 (0.95, 1.37)	0.16	1.08 (0.88, 1.32)	0.48
**Medium/Dry**	1.08 (0.76, 1.54)	0.66	1.04 (0.75, 1.43)	0.82	0.89 (0.62, 1.26)	0.50
**Interaction**	1.06 (0.78, 1.43)	0.71	0.91 (0.70, 1.19)	0.50	0.82 (0.62, 1.10)	0.19
**Number of Pathogens**						
**Wet**	-0.17 (-0.45, 0.11)	0.24	0.01 (-0.25, 0.27)	0.94	0.04 (-0.25, 0.32)	0.80
**Medium/Dry**	-0.08 (-0.56, 0.41)	0.76	-0.14 (-0.54, 0.27)	0.50	-0.08 (-0.57, 0.41)	0.75
**Interaction**	0.09 (-0.30, 0.49)	0.65	-0.15 (-0.46, 0.17)	0.36	-0.12 (-0.51, 0.28)	0.56
**EAEC**						
**Wet**	0.91 (0.70, 1.19)	0.51	0.95 (0.72, 1.25)	0.71	1.22 (0.93, 1.61)	0.14
**Medium/Dry**	1.22 (0.72, 2.06)	0.45	0.89 (0.56, 1.39)	0.60	1.05 (0.65, 1.69)	0.84
**Interaction**	1.34 (0.85, 2.10)	0.21	0.93 (0.65, 1.33)	0.70	0.86 (0.58, 1.27)	0.45
**DAEC**						
**Wet**	0.92 (0.81, 1.06)	0.24	0.99 (0.86, 1.13)	0.85	1.06 (0.93, 1.22)	0.38
**Medium/Dry**	0.92 (0.72, 1.17)	0.50	1.10 (0.87, 1.37)	0.43	0.81 (0.63, 1.02)	0.08
**Interaction**	1.00 (0.81, 1.22)	0.97	1.11 (0.93, 1.33)	0.26	0.76 (0.62, 0.92)	0.01
**aEPEC**						
**Wet**	1.01 (0.73, 1.40)	0.96	1.20 (0.88, 1.64)	0.26	1.08 (0.70, 1.65)	0.74
**Medium/Dry**	1.17 (0.64, 2.14)	0.62	0.87 (0.51, 1.5)	0.62	0.89 (0.46, 1.73)	0.74
**Interaction**	1.16 (0.70, 1.92)	0.57	0.73 (0.47, 1.14)	0.16	0.83 (0.50, 1.37)	0.47

**tEPEC**						
**Wet**	0.89 (0.59, 1.32)	0.55	1.00 (0.61, 1.64)	1.00	0.97 (0.55, 1.73)	0.92
**Medium/Dry**	0.79 (0.35, 1.82)	0.59	0.84 (0.34, 2.06)	0.70	1.09 (0.45, 2.67)	0.85
**Interaction**	0.90 (0.43, 1.86)	0.77	0.84 (0.40, 1.78)	0.65	1.12 (0.57, 2.23)	0.74
**ETEC**						
**Wet**	0.79 (0.31, 2.05)	0.63	0.77 (0.33, 1.81)	0.55	1.53 (0.63, 3.70)	0.35
**Medium/Dry**	1.06 (0.21, 5.27)	0.95	0.21 (0.03, 1.35)	0.10	0.93 (0.22, 3.98)	0.92
**Interaction**	1.33 (0.36, 4.88)	0.67	0.27 (0.05, 1.42)	0.12	0.61 (0.19, 1.93)	0.40
**Shigella**						
**Wet**	0.90 (0.52, 1.56)	0.71	0.65 (0.4, 1.06)	0.08	1.06 (0.63, 1.79)	0.82
**Medium/Dry**	0.86 (0.33, 2.25)	0.76	0.97 (0.37, 2.54)	0.95	0.93 (0.38, 2.28)	0.87
**Interaction**	0.95 (0.43, 2.10)	0.90	1.5 (0.65, 3.43)	0.34	0.88 (0.42, 1.81)	0.72
**Campylobacter**						
**Wet**	0.82 (0.51, 1.32)	0.42	1.32 (0.87, 2.01)	0.19	1.08 (0.69, 1.70)	0.73
**Medium/Dry**	0.85 (0.36, 1.99)	0.70	1.04 (0.53, 2.03)	0.90	0.83 (0.39, 1.76)	0.63
**Interaction**	1.03 (0.50, 2.09)	0.94	0.79 (0.47, 1.33)	0.37	0.77 (0.42, 1.4)	0.38
**Norovirus**						
**Wet**	2.26 (1.22, 4.21)	0.01	1.08 (0.63, 1.85)	0.79	0.67 (0.26, 1.74)	0.41
**Medium/Dry**	1.49 (0.54, 4.08)	0.44	1.46 (0.54, 3.92)	0.45	0.91 (0.19, 4.41)	0.90
**Interaction**	0.66 (0.30, 1.46)	0.30	1.36 (0.59, 3.11)	0.47	1.35 (0.38, 4.78)	0.64
**Cryptosporidium**						
**Wet**	0.64 (0.33, 1.23)	0.18	1.60 (0.86, 2.99)	0.14	1.34 (0.76, 2.34)	0.31
**Medium/Dry**	0.74 (0.28, 1.93)	0.53	1.25 (0.45, 3.50)	0.67	0.91 (0.32, 2.57)	0.86
**Interaction**	1.16 (0.57, 2.32)	0.69	0.78 (0.35, 1.77)	0.56	0.68 (0.28, 1.63)	0.39
**Giardia**						
**Wet**	1.13 (0.62, 2.08)	0.69	1.10 (0.68, 1.77)	0.71	0.87 (0.51, 1.47)	0.60
**Medium/Dry**	1.11 (0.38, 3.19)	0.85	1.11 (0.44, 2.80)	0.83	0.97 (0.40, 2.40)	0.95
**Interaction**	0.98 (0.41, 2.33)	0.96	1.01 (0.46, 2.23)	0.98	1.12 (0.54, 2.34)	0.76

Antecedent conditions were calculated using the sum of total rainfall over the 8 weeks prior to sample collection, where wet conditions are those exceeding 67th percentile for total rainfall over the 8-week period compared to the study period and medium/dry conditions are those below the 67th percentile for total rainfall. HREs were defined as a day where the total rainfall was above the 95th percentile (8.81mm) for the overall study period. All models adjusted for rolling mean temperature during the same period, intervention status, access to a direct household connection to a piped water source, poverty, caregiver education level, caregiver employment status, and basic sanitation access.
