# Supplementary material for: Variability of enteric pathogen infections by season and meteorological conditions in a low-income, urban setting in Mozambique
Source: PLOS Glob Public Health. 2026 Apr 28;6(4):e0005330. doi: 10.1371/journal.pgph.0005330 (PMC13123936; doi:10.1371/journal.pgph.0005330)
Supplement: S5 Table — (PDF) [file pgph.0005330.s006.pdf]

**S5 Table.** Adjusted associations of above vs. below median temperatures on enteric pathogen infections.

| | **0-1 week before sample** | | **1-2 weeks before sample** | | **2-3 weeks before sample** | |
|---|---|---|---|---|---|---|
| | **aPR or aβ (95% CI)** | ***p-value*** | **aPR or aβ (95% CI)** | ***p-value*** | **aPR or aβ (95% CI)** | ***p-value*** |
| **Combined outcomes** | | | | | | |
| **Any bacteria** | 1.00 (0.91, 1.10) | 0.99 | 0.98 (0.89, 1.07) | 0.61 | 0.99 (0.90, 1.10) | 0.91 |
| **Any protozoa** | 0.62 (0.47, 0.80) | <0.01 | 0.65 (0.49, 0.86) | <0.01 | 0.62 (0.48, 0.79) | 0.00 |
| **Any virus** | 0.96 (0.76, 1.23) | 0.77 | 0.94 (0.74, 1.19) | 0.62 | 1.05 (0.82, 1.34) | 0.70 |
| **Co-infection** | 0.91 (0.80, 1.04) | 0.17 | 0.86 (0.76, 0.97) | 0.02 | 0.92 (0.81, 1.03) | 0.16 |
| **Number of infections** | -0.11 (-0.33, 0.11) | 0.33 | -0.13 (-0.33, 0.07) | 0.19 | -0.14 (-0.36, 0.08) | 0.20 |
| **Bacterial outcomes** | | | | | | |
| **EAEC** | 1.08 (0.92, 1.26) | 0.34 | 1.08 (0.91, 1.29) | 0.36 | 1.16 (0.96, 1.40) | 0.13 |
| **DAEC** | 0.94 (0.85, 1.03) | 0.19 | 0.89 (0.80, 0.98) | 0.01 | 0.91 (0.82, 1.01) | 0.07 |
| **tEPEC** | 1.15 (0.82, 1.62) | 0.41 | 1.10 (0.77, 1.58) | 0.59 | 1.07 (0.76, 1.50) | 0.72 |
| **aEPEC** | 0.93 (0.76, 1.14) | 0.49 | 0.87 (0.71, 1.07) | 0.19 | 0.93 (0.76, 1.13) | 0.46 |
| **ETEC** | 1.49 (0.92, 2.42) | 0.11 | 1.45 (0.86, 2.43) | 0.16 | 1.22 (0.72, 2.07) | 0.46 |
| **Shigella** | 1.23 (0.80, 1.90) | 0.35 | 1.24 (0.79, 1.94) | 0.35 | 1.21 (0.79, 1.87) | 0.38 |
| **Campylobacter** | 1.01 (0.74, 1.38) | 0.96 | 0.91 (0.70, 1.18) | 0.46 | 1.00 (0.77, 1.30) | 1.00 |
| **Viral outcomes** | | | | | | |
| **Norovirus** | 1.44 (0.89, 2.33) | 0.13 | 1.41 (0.89, 2.21) | 0.14 | 1.65 (1.08, 2.54) | 0.02 |
| **Protozoan infections** | | | | | | |
| **Cryptosporidium** | 0.43 (0.27, 0.68) | <0.01 | 0.40 (0.27, 0.59) | <0.01 | 0.38 (0.27, 0.54) | <0.01 |
| **Giardia** | 0.77 (0.56, 1.07) | 0.12 | 0.93 (0.64, 1.37) | 0.72 | 0.87 (0.62, 1.20) | 0.39 |

Above median temperatures were defined as rolling average weekly temperature above the 50th percentile (25.0°C) for the full study period. All models adjusted for rolling mean precipitation during the same period, intervention status, access to a direct household connection to a piped water source, poverty, caregiver education level, caregiver employment status, and basic sanitation access. Models only run for enteric infections with prevalence over 10%.
