# Supplementary material for: Variability of enteric pathogen infections by season and meteorological conditions in a low-income, urban setting in Mozambique
Source: PLOS Glob Public Health. 2026 Apr 28;6(4):e0005330. doi: 10.1371/journal.pgph.0005330 (PMC13123936; doi:10.1371/journal.pgph.0005330)
Supplement: S6 Table — (PDF) [file pgph.0005330.s007.pdf]

S6 Table. Adjusted associations of above vs. below 33rd tertile temperatures on enteric pathogen infections.

	**0-1 week before sample**		**1-2 weeks before sample**		**2-3 weeks before sample**	
	**aPR or aβ (95% CI)**	***p-value***	**aPR or aβ (95% CI)**	***p-value***	**aPR or aβ (95% CI)**	***p-value***
**Combined outcomes**						
**Any bacteria**	1.02 (0.94, 1.12)	0.59	0.98 (0.89, 1.07)	0.65	0.99 (0.90, 1.10)	0.91
**Any protozoa**	1.47 (1.16, 1.85)	0.00	1.42 (1.12, 1.80)	0.00	1.43 (1.09, 1.87)	0.01
**Any virus**	0.91 (0.74, 1.12)	0.35	0.83 (0.65, 1.06)	0.13	0.93 (0.73, 1.18)	0.54
**Co-infection**	1.16 (1.02, 1.31)	0.02	1.09 (0.96, 1.23)	0.18	1.13 (0.98, 1.30)	0.09
**Number of infections**	0.15 (-0.04, 0.35)	0.12	0.11 (-0.09, 0.32)	0.27	0.12 (-0.11, 0.35)	0.30
**Bacterial outcomes**						
**EAEC**	0.92 (0.75, 1.12)	0.41	0.84 (0.70, 1.00)	0.05	0.94 (0.78, 1.13)	0.47
**DAEC**	1.04 (0.94, 1.14)	0.48	1.09 (1.01, 1.19)	0.03	1.09 (0.99, 1.18)	0.07
**tEPEC**	0.79 (0.57, 1.09)	0.15	0.82 (0.59, 1.16)	0.27	0.75 (0.53, 1.04)	0.08
**aEPEC**	1.02 (0.85, 1.23)	0.83	1.13 (0.91, 1.41)	0.26	1.14 (0.92, 1.43)	0.24
**ETEC**	0.87 (0.52, 1.45)	0.59	0.73 (0.43, 1.24)	0.24	0.76 (0.43, 1.33)	0.33
**Shigella**	1.27 (0.83, 1.94)	0.26	0.98 (0.62, 1.54)	0.92	0.93 (0.57, 1.52)	0.76
**Campylobacter**	1.09 (0.84, 1.41)	0.52	1.03 (0.81, 1.31)	0.80	0.94 (0.75, 1.19)	0.61
**Viral outcomes**						
**Norovirus**	0.47 (0.27, 0.83)	0.01	0.51 (0.28, 0.93)	0.03	0.64 (0.37, 1.11)	0.11
**Protozoan infections**						
**Cryptosporidium**	1.99 (1.41, 2.82)	0.00	1.91 (1.35, 2.71)	0.00	1.90 (1.34, 2.70)	0.00
**Giardia**	1.19 (0.87, 1.62)	0.28	1.11 (0.77, 1.60)	0.58	1.13 (0.80, 1.60)	0.48

Below 33rd percentile temperatures were defined as rolling average weekly temperature below the 33rd percentile (23.3ºC) for the full study period. All models adjusted for rolling mean precipitation during the same period, intervention status, access to a direct household connection to a piped water source, poverty, caregiver education level, caregiver employment status, and basic sanitation access.
